# Whole-body isometric force/torque measurements for functional assessment in neuro-rehabilitation: platform design, development and verification

**DOI:** 10.1186/1743-0003-6-38

**Published:** 2009-10-30

**Authors:** Stefano Mazzoleni, Andras Toth, Marko Munih, Jo Van Vaerenbergh, Giuseppe Cavallo, Silvestro Micera, Paolo Dario, Eugenio Guglielmelli

**Affiliations:** 1 ARTS Lab, Scuola Superiore Sant'Anna, Pisa, Italy; 2 Department of Manufacturing Engineering, Budapest University of Technology and Economics, Budapest, Hungary; 3 Laboratory of Robotics and Biomedical Engineering, Faculty of Electrical Engineering, University of Ljubljana, Ljubljana, Slovenia; 4 Laboratorium for Neuro- and Psychophysiology, Katholieke Universiteit Leuven, Belgium; 5 Laboratory of Biomedical Robotics & EMC, Università Campus Bio-Medico, Rome, Italy

## Abstract

**Background:**

One of the main scientific and technological challenges of rehabilitation bioengineering is the development of innovative methodologies, based on the use of appropriate technological devices, for an objective assessment of patients undergoing a rehabilitation treatment. Such tools should be as fast and cheap to use as clinical scales, which are currently the daily instruments most widely used in the routine clinical practice.

**Methods:**

A human-centered approach was used in the design and development of a mechanical structure equipped with eight force/torque sensors that record quantitative data during the initiation of a predefined set of Activities of Daily Living (ADL) tasks, in isometric conditions.

**Results:**

Preliminary results validated the appropriateness, acceptability and functionality of the proposed platform, that has become now a tool used for clinical research in three clinical centres.

**Conclusion:**

This paper presented the design and development of an innovative platform for whole-body force and torque measurements on human subjects. The platform has been designed to perform accurate quantitative measurements in isometric conditions with the specific aim to address the needs for functional assessment tests of patients undergoing a rehabilitation treatment as a consequence of a stroke.

The versatility of the system also enlightens several other interesting possible areas of application for therapy in neurorehabilitation, for research in basic neuroscience, and more.

## Background

One of the main scientific and technological challenges of rehabilitation bioengineering is the development of innovative methodologies, based on the use of appropriate technological devices, for the assessment of the motor output from patients undergoing a rehabilitation treatment. Such tools should be as fast and cheap to use as clinical scales, which are currently the daily instruments most widely used in the routine clinical practice.

In this work, our interest is focused on the development of mechatronic platform for the assessment of stroke patients using force/torque (F/T) measurements in isometric conditions.

Stroke is the third leading cause of death in most developed countries [1] and the leading cause of long term disability [2,3].

During the last decade, different research studies focused on the identification of milestones characterizing the motor rehabilitation process [4] and predictive markers of functional recovery from stroke [5].

A measure of functional abilities may be obtained by using functional assessment scales that include the ability to perform Activities of Daily Living (ADL). These scales are commonly used to monitor the patients' progress [6-8], but present several limitations due to their subjective nature. The development of methods and devices for objective measures of patients' motor output represent a need in the rehabilitation domain.

In the last decade, different types of dynamometer have been developed, but only few simple devices for isometric measurements came on to the market.

Different devices have been already developed: commercially available dynamometers equipped with force/torque (F/T) sensors are able to record the forces and torques that are produced at contact points between the human body and the environment. From the biomechanical point of view, they provide information on the joint forces and torques exerted by the patient., but show three main limitations: they (i) impose artificial motions or loadings on the subject, (ii) can measure only one degree-of-freedom (DOF) F/T data at the same time and (iii) provide only a little information about the underlying physiological and neural mechanisms.

In order to overcome the above mentioned drawbacks, our approach focuses on the changes in repetitively measured F/T patterns recorded during the initiation of different ADL tasks using isometric contractions: our hypothesis is that a mechatronic device based on such approach can track the course of recovery in stroke and thus determine the effectiveness of applied rehabilitation treatments.

Recently, the application of robotics and mechatronic technology to functional assessment and motor therapy has been introduced in neurological rehabilitation [9,10] with very encouraging early clinical results [11].

To date, the use of voluntary isometric contractions for assessment purposes in humans was applied to single joints [12,13] and groups of muscles [14].

The neurophysiological assumptions which inspired the motivations for the design and the development of the proposed mechatronic platform rely on the recent neuroscientific findings about the forward model as internal representation of the causal relationship between sensorimotor signals and motor commands [15-18].

In the first days after a stroke, the amplitude of any possible movement is very limited: the basic idea is to perform isometric measurement of functional directed movements. The choice of using an isometric approach is based on the discovery that imagination and initiation of the task have the same functional properties as performing the task [19-26].

Some authors have proposed the use of isometric forces and torques for the analysis of the intention to perform a given task [27]. This approach can detect some sensorimotor representations in the early phases after a stroke [28,29]. This methodology, based on the movement initiation, can allow the verification of the existence of a still unimpaired or damaged forward model for a specific functional task. This model maps motor commands onto their corresponding sensory consequences and will adapt itself according to new dynamical situations. After a stroke, in addition to the reduced or lost neuromuscular voluntary activation, different sensorimotor functions may be impaired: spasticity, co-contraction and muscle synergies as well as compensation may interfere with the quality of the motor tasks.

The parameters which measure the possible motor recovery could be implemented in order to reveal day by day changes in the motor system.

The forward model can be preserved, but the motor output is reduced or disorganized, either may show modifications through an adapted motor output. It can happen that the forward model fails or is even abolished, leading to negative consequences for the movements smoothness [26,30].

The possible improvement of functional performance can be assessed using ADL tasks, which represent motor routines with associated defined forward models. Moreover, their motor patterns are expected to be highly repeatable and predictable.

Since the main objective of our approach is to find a quantitative functional assessment during rehabilitation, the implementation of synchronous multichannel F/T data recordings is essential.

In this paper a diagnostic device which has the capability of measuring multichannel F/T data in isometric conditions from different body locations, starting from natural positions is presented. There are no similar commercially available isokinetic or isometric devices on the market that meet previous requirements.

Therefore, the diagnostic device here presented represents an original contribution to the modern challenges of functional assessment of motor recovery to be used in neurorehabilitation clinical practice.

A human-centered design methodology, which will be presented in the next section together with the functional specifications, was chosen as methodological approach for the development of the proposed diagnostic device.

In conclusion, the research presented in the paper was aimed at developing a diagnostic device and an innovative protocol for the functional assessment of post-stroke patients, based on multichannel F/T measurements on the whole body, recorded during ADLs tasks in isometric conditions.

## Methods

### Basic requirements

The neuroscientific findings described in the previous section form the basis for the design of an innovative diagnostic device. An interactive design process involved rehabilitation specialists and engineers toward the definition of basic requirements for such device:

• capability of recording F/T data from the hand, from the arm, the trunk, the seat, and from the foot of a patient seated on a standard wheelchair;

• easy and quick adjustments for the different anthropometrical characteristics of the population;

• to be used on both the right and left body side;

• requiring a minimum physical effort and time to the operator;

• capability of recording measurements in three different postures;

• to be a modular system.

### Functional specifications

The main objective of recording isometric F/T measurements is to obtain quantitative assessment during stroke rehabilitation. Every isometric measurement is used to determine the actual motor status of the patient. A detailed clinical protocol was developed in order to perform F/T measurements on post-stroke patients [31].

Six different ADL tasks with a varying complexity were used for such purpose. An initial list of 42 possible tasks were selected consulting reference textbooks [32-35]. Because of time constraints (30 minutes for each measuring cycle), six tasks were finally selected from the 42 formerly proposed. Each task demonstrates features of a reached functional milestone during the recovery from stroke.

Post-stroke patients have been invited to perform them in a prescribed order. For each task, five different recordings were performed: on a monitor placed in front of him/her, the patient watches a video showing the movement associated to each ADL task to be performed (recording #1). Secondly he is asked to mentally imagine and reproducing it with open eyes (recording #2). Finally, after the appearance of a green light on the monitor, he is asked to repeat the task for three times, trying to exert the forces at a comfortable level (recording #3, recording #4 and recording #5).

On the basis of the measuring protocol, the following functional specifications were chosen. Patients were seated in a special designed wheelchair and driven into an anthropometrical adaptive measuring instrument. Appropriate size accessories and device settings were also used to ensure that the error in the anatomical angles is minimal, as well as to keep the handling complexity of the diagnostic device on a tolerable level for the operating physiotherapist.

Three different positional settings, described in the following section, were chosen for the measurements: they represent a trade-off between a good approximation of natural postures and the anthropometric characteristics of the subject. This choice assures sufficient conditions of repeatability to the measurements.

A clinical assessment was performed through the Fugl-Meyer Scale (Lindmark adaptation), the Motor Assessment Scale and the Stroke Impact Scale. The physiotherapists used a Portable Digital Assistant (PDA) in order to record the scores for each assessment scale and patients' functional recovery by using natural language descriptions.

To assure an high reproducibility during the entire period of data acquisition in clinical trials it was necessary to measure a large number of patients with the same device and in the same anatomical starting position.

The data acquisition system has recorded isometric F/T data from:

• the trunk (at the patient's back),

• the lower trunk (at the patient's fundament: the corresponding F/T sensor is placed under the wheelchair's seat plate),

• the impaired lower limb,

• the impaired foot and toe,

• the impaired middle finger, index finger and thumb.

ADL tasks to be performed during isometric F/T measurements are listed in the following sequence, together with the corresponding object:

#### ADL task #1

Drinking a glass (no reaching): the arm is placed close to the body, close to the mid line, the position of the foot is standard, the fingers of the hand are prepared for a cylindrical grasp. Object: glass placed close to the hand.

#### ADL task #2

Turning a key: the starting position is the same as for grasping the glass. Object: a key in a lock located in front of the hand. The key should be oriented horizontally in the lock.

#### ADL task #3

Taking a spoon: the starting position is the same as for grasping the glass. The reaching movement towards the spoon is measured. The position of the foot is standard. Object: a spoon is placed a bit higher than the glass, on the side of the back of the hand.

#### ADL task #4

Lifting a bag: the starting position of the arm is at the side of the body, the elbow is in a natural position (slightly flexed), the position of the hand and the foot are standard, the fingers of the hand are prepared for a cylindrical grasp. Object: a bag placed on the ground.

#### ADL task #5

Reaching for a bottle: the starting position is an almost extended arm over the midline. The starting position of the hand is the same as for drinking the glass. The position of the foot is slid backward, and the back should be leaned forward. Object: a bottle placed in front of the hand.

#### ADL task #6

Bringing the bottle to the other side: the starting positions of the arm, hand, and the foot are the same as for reaching for a bottle. Object: a bottle placed in front of the affected upper limb at arm reach distance.

As mentioned earlier, the platform has three positional settings for the patient according to the six selected ADL tasks to be performed during isometric F/T measurements: position 1 covers ADL task #1, ADL task #2 and ADL task #3, position 2 is related to ADL task #4, and position 3 covers ADL task #5 and ADL task #6.

The above functional specifications have been included into the design methodology presented in the following section.

### Human-centered design methodology

A human-centered mechatronic design approach has been followed by starting from anthropometrical considerations and iteratively refined in a tight debate with clinicians and end-users (i.e., therapists, patients). Simulations, mock-ups and two different prototypes of the platform have been extensively used to obtain direct feedbacks from end-users and to enable experimental preliminary tests in the real application domain.

The proposed method for isometric F/T measurements requires fixed, very stiff, anatomically standard and, at the same time, repeatable individual setting of the device for each patient in order to ensure reproducibility, reliability and good precision in the isometric measurements.

Design requirements of the platform arose from three different areas. Firstly, standardisation of the measurement, secondly safety standards, as well as medical certification requirements. Finally, space limitations in hospitals regarding the room where the device was used and the location where the wheelchairs was stored when they are not in use, were taken into account. Standardisation of the postures and measurement procedure, including a calibration routine, assured reliability and validity to the recorded F/T measurements.

As a reference position, the user is seated on a wheelchair at height of 580 mm from the floor of the platform and whose back is 330 mm back from the rear side of the device.

**Figure 1:**
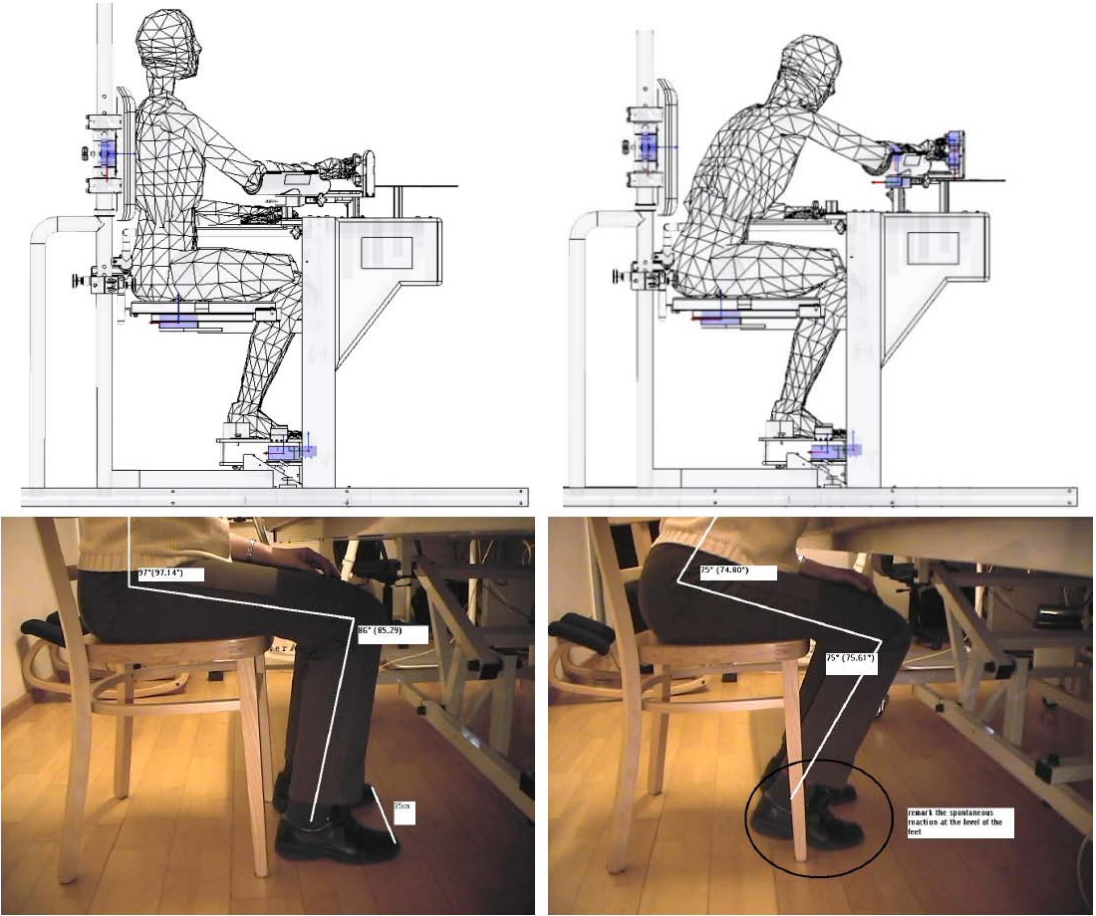
**Sample CAD models used in the ergonomic study (top) and the two corresponding selected postures for lower extremities and feet (bottom)**.

In this configuration, isometric contractions in two reference postures of the lower extremities can be performed by using the proposed platform (Figure 1). In the former, the user is seating in a neutral posture.

This position is typical for the initiation of most common tasks, such as lifting or grasping an object: it is the starting position for ADL task #1, ADL task #2, ADL task #3 and ADL task #4. The latter takes into account a different posture which enables the user to move the trunk forward and the feet backward. Starting from this position, other tasks, such as a forward reaching tasks, can be performed: it is the starting position for ADL task #5 and ADL task #6.

The anthropometrical data of the European population was studied [36]. The ergonomic study was performed through CAD simulations (Pro/Engineer): a 3D mannequin model, created by using the Mannequin Pro tool, has been inserted into the CAD environment with the aim of (i) simulating the different postures according to the gender and percentile and (ii) fitting the design of the platform to the anatomical positions accordingly.

The results of this study enlightened the possibility of implementing a limited number of discrete settings on the platform, henceforth named S (Small), M (Medium) and L (Large), corresponding to the values of the 25th percentile female (S size), the mean of the 50th percentile male and 50th percentile female (M size), and the 75th percentile male (L size).

Therefore, the device can be set without error to the above mentioned percentiles of the population, which represents a vast majority of the population.

The adjustability of the device to the three discrete patient sizes was implemented. To minimize the error in the anatomical angles to be set at each of the six ADLs, as well as to keep the handling complexity of the diagnostic device on a tolerable level for the operating physiotherapist, the patients recruited for the isometric F/T measurements were classified into three groups according to their height. During the measurements the appropriate size accessories and device settings were used. The size groups were denoted by the S, M, L labels and colour codes were used in order to make the operations easier (Table 1).

**Table 1:** Definition of patients' division into three groups, according to their height.

**Label**	**Colour code**	**Height (h)**
S	Yellow	h < 1625 mm

M	Green	1625 mm < h < 1751 mm

L	Blue	h > 1751 mm

As a consequence of the mentioned approach, the set of the anatomical angles in position 1, position 2 and position 3 was fixed for any patient size. These angles, except those in neutral positions are listed in Table 2. The calculated deviation from the ideal anatomical angles remained in the range ± 0.5°.

**Table 2:** The values of anatomical angles in Position 1, Position 2 and Position 3.

**Articular movement**	**Position 1**	**Position 2**	**Position 3**
Shoulder abduction	15	5	0

Shoulder flexion	50	0	100

Shoulder extension	0	7	0

Shoulder internal rotation	45	0	45

Elbow flexion	35	12	20

Thumb abduction	50	50	50

Finger metacarpophalangeal flexion	15	20	15

Finger proximal interphalangeal flexion	20	0	20

Finger distal interphalangeal flexion	20	20	20

Lumbar-thoracic flexion	0	0	30

Lumbar-thoracic rotation	0	0	20

Lumbar-thoracic lateral flexion	0	0	18

Hip flexion	90	90	90

Knee flexion	90	90	110

Ankle dorsiflexion	0	0	8

Toe metatarsophalangeal flexion	0	0	7

The anthropometrical and ergonomic design approach, by identifying only a limited number of adjustments required to the therapist, clearly simplified the design and the development of the overall system, presented in the following section, and represents an advantage in terms of setup time.

### The Alladin Diagnostic Device (ADD)

The design choice was to develop a simple and low cost platform for clinical applications: in principle, every degree of freedom in the platform can be actuated for the adjustments and for transforming it into a robotic system, if necessary. This could be done without altering radically the present design.

The main objective of the mechatronic platform, henceforth named Alladin Diagnostic Device (ADD), here presented is to perform valid and reliable isometric F/T measurements at stroke patients during the execution of the six ADL tasks. The cycle time for a single isometric force torque measurements (including measurement of the six ADL tasks) was 30 minutes. No significant time difference between healthy controls and patients was found. The ADD has to provide repeatable and accurate measurements: given this important requirement, the patients were precisely positioned to the same set of ADL positions for each recording.

The standardization achieved both in terms of the mechanics of the device, the F/T sensor unit, the measurement control software and the unambiguous guidelines on the operation of the device have resulted in high reproducibility and comparability of the F/T measurements.

Since April 2004, a complete product design and development cycle, including a computer aided design, the development of three early prototypes and the feedback from the testing, were implemented. Refinement and detailing of the conceptual design was a natural result of this cyclic process.

The choice of the sensors was leaded by the measurement input ranges which was derived partly on the basis of existing references about typical data on human subjects [37-41], and partly on the basis of preliminary measurements. Eight 6-axis F/T commercial sensors were respectively installed behind the trunk, below the posterior, at the affected lower arm, at the affected thumb, index and middle finger, at the affected foot and toe, output detailed data on the ADL tasks to be performed. Table 3 shows the basic characteristics of the 6-axis F/T sensors (50M31A-I25, 67M25A-I40, 90M40A-I50, 45E15A-U760, JR3 Inc., Woodland, USA).

**Table 3:** 6-axis F/T sensors: basic characteristics.

**Qty**	**Model**	**Description**	**Lateral forces (Fx, Fy) [N]**	**Axial force (Fz) [N]**	**Torques (Tx, Ty, Tz) [Nm]**	**Dimensions [mm]**
3	50M31A-I25 150N8	Type-H(and)	150	300	8	Ø 50 × 31
1	67M25A-I40 150N10	Type-A(rm)	150	200	10	Ø 67 × 35
1	90M40A-I50 250N20	Type-B(ack)	250	250	20	Ø 90 × 40
1	45E15A-U760 1200N120	Type-S(eat)	600	1200	120	Ø 114 × 40
1	90M40A-I50 400N25	Type-F(oot)	400	800	25	Ø 90 × 40
1	50M31A-I25 150N8	Type-T(oe)	150	300	8	Ø 50 × 31

**Figure 2:**
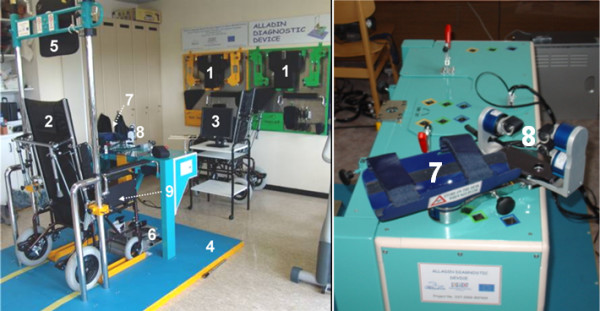
**The components of the platform: 1) Accessory storage board, 2) Transit lying wheelchair, 3) Monitor for the patient, 4) Podium, 5) Trunk Device, 6) Foot Device, 7) Arm Device, 8) Finger Device, 9) Seat Device**.

All operating instructions are presented on a screen in front of the patient. The combined output of 48 channels representing the x, y, z components of F/T signals for all eight sensors are recorded using a sampling frequency of 100 Hz. The diagnostic device includes the following main units (Figure 2):

1. Accessory storage board

2. Transit lying wheelchair

3. Monitor for the patient

4. Podium

5. Trunk Device

6. Foot Device

7. Arm Device

8. Finger Device

9. Seat Device

**Figure 3:**
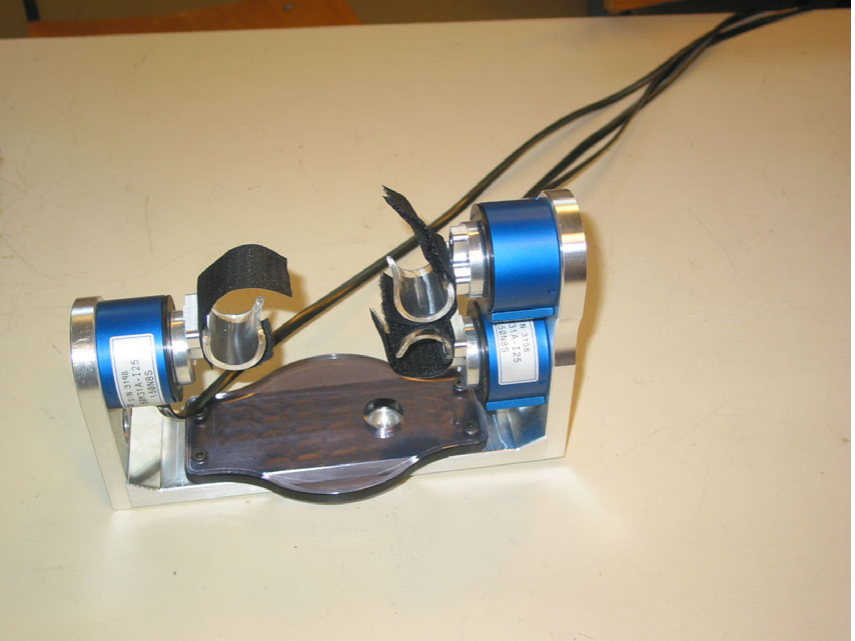
The Finger Device.

**Figure 4:**
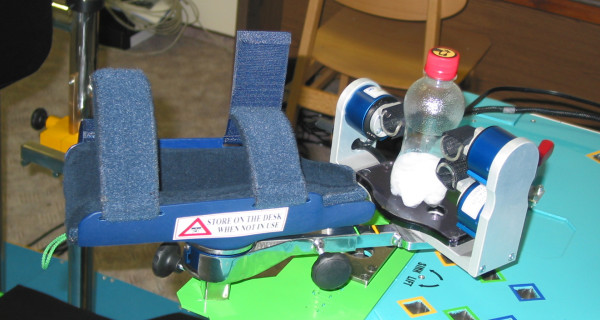
The Arm Device.

**Figure 5:**
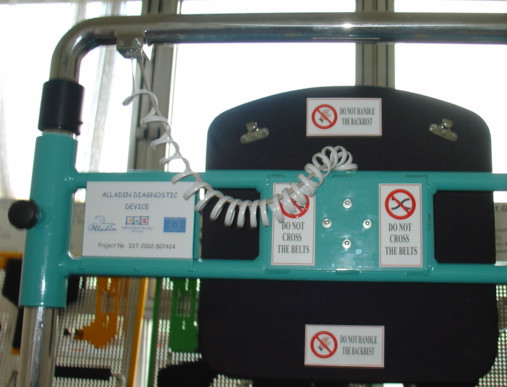
The Trunk Device.

**Figure 6:**
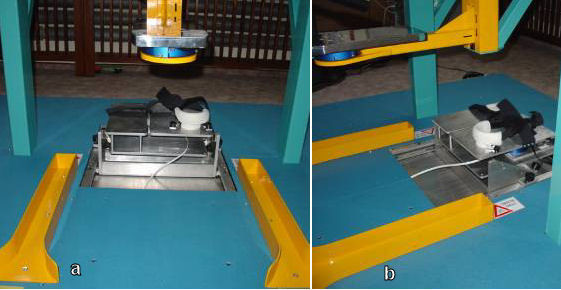
The Seat Device: rear view (a), lateral view (b).

**Figure 7:**
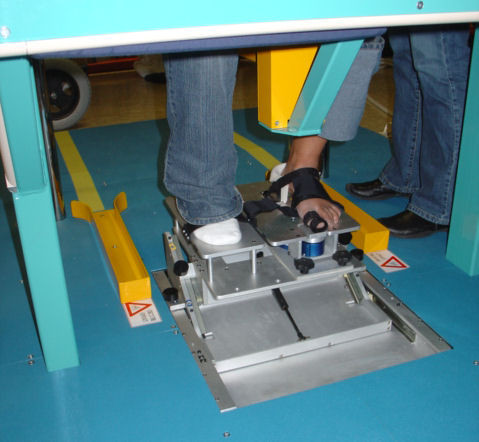
**The Foot Device**.

The Finger Device, the Arm Device, the Trunk Device, the Seat Device and the Foot Device are shown in Figure 3, 4, 5, 6 and 7 respectively. A customized software has been developed in order to manage all the functionalities provided by the ADD, including the recording and exchange of different types of data between the different modules. The main data to be collected and managed are:

• Patient data and case history

• Standard Outcome Measure (SOM)

• Natural language descriptions of the patient's status

• Voice records of the descriptions

• F/T measurement records from ADL tasks

**Figure 8:**
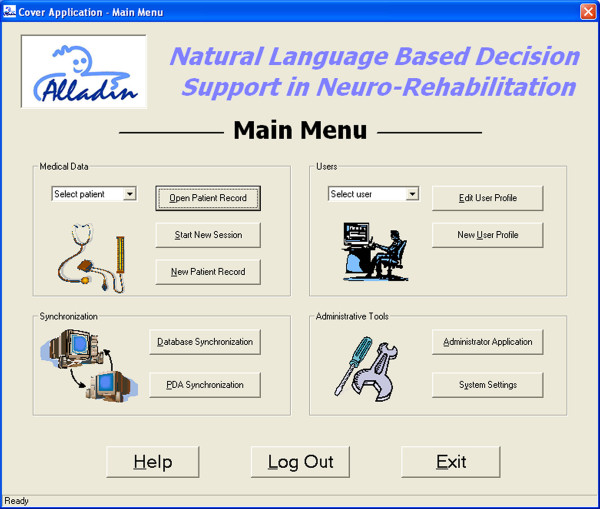
The main menu of the graphical user interface.

All data, after having been collected, are uploaded to a local database. The graphical user interface (Figure 8) offers different functionalities, such opening a patient record, starting a new session of measurements, creating a new patient record, editing and creating an user's profile, synchronizing with the global DB, system settings adjustment and remote assistance. Four different types of users were identified (ADD physiotherapist, Natural language physiotherapist, Principal Investigator and System administrator): for each user profile an access rights policy was defined.

Different tests were performed in order to investigate the measurements characteristics of the device: time and frequency analysis of the force series and their derivatives led to the application of appropriate pre-processing data filtering [42]

Several young volunteers participated in a preliminary testing aimed at verifying the output of the proposed isometric procedure and improving the design choices.

## Results

The results from the clinical trials, started in February 2005 and ended in September 2006, whose report is not the aim of this paper, demonstrate that the device performs all operations in accordance with system requirements and functional specifications.

Patients were recruited following these inclusion criteria: 1) hemiparesis due to stroke, 2) the brain impairment must be provided by CT or MRI, 3) minimum age is 18 years, 4) the subject must be suitable to endure physical load during the measurements, 5) the subject must be cooperative, 6) signed informed consent. Exclusion criteria were: 1) restricted disposing capacity or legal capacity, 2) prisoner, 3) movement or other disorder that makes it impossible for the patient to sit calmly during the treatment, 4) skin problem where use of an orthosis is contra-indicated, 5) patients shorter than 1530 mm and taller than 1870 mm, 6) patients with a weight over 110 kg.

The centres participating in the multi-centre clinical trials were:

• Algemeen Ziekenhuis Maria Middelares Sint-Jozef Hospital (AZMMSJ), Gent, Belgium

• Adelaide & Meath Hospital (AMNCH), Tallaght, Dublin, Ireland

• Szent János Hospital, Budapest, Hungary

All three clinical centres obtained the approval of the relevant ethics committees for performing the trials. An informed consent was obtained from each participating subject, who was measured and assessed twice a week for the first two months period and once a week during four consecutive months. No adverse events occurred during the trials.

Table 4 shows the comparison among the scores of Fugl-Meyer (Hand section), Motor Assessment Scale ("Hand movements" and "Advanced Hand Activities" sections), Stroke Impact Scale (Hand function) scores and the mean value of force resultant vector in three hand sensors (Thumb, Index, Middle Finger), "Drinking" task, attempt #4, from 10 post-stroke patients.

**Table 4:** Comparison among Fugl-Meyer (Hand section), Motor Assessment Scale ("Hand movements" and "Advanced Hand Activities"), Stroke Impact Scale (Hand function) scores and mean value of force resultant vector from three hand sensors (Thumb, Index, Middle Finger), "Drinking" task, attempt #4, in ten post-stroke patients.

**Subject**	**Week**	**Mean [N]**	**FM**^1^	**MAS**^2^	**SIS**^3^
**Subject_1 TCD-063**	**2**	**4.28296**	**17**	**10 **(s05)	**N/A**
	
	**27**	**4.31637**	**21**	**11 **(s31)	**16 **(s16)
	
	**37**	**4.52127**	**19**	**11 **(s41)	**16 **(s41)

**Subject_2 TCD-067**	**3**	**12.2230**	**14**	**9 **(s05)	**20 **(s05)
	
	**9**	**15.2048**	**17**	**12 **(s11)	**N/A**
	
	**27**	**25.7825**	**21**	**12 **(s29)	**5 **(s05)

**Subject_3 AHS-072**	**8**	**1.99971**	**17**	**8 **(s10)	**5 **(s08)
	
	**16**	**1.23859**	**18**	**9 **(s20)	**5 **(s16)
	
	**27**	**4.25887**	**18**	**9 **(s30)	**5 **(s27)

**Subject_4 AHS-023**	**3**	**8.493**	**20**	**8 **(s07)	**5 **(s03)
	
	**13**	**15.799**	**19**	**7 **(s16)	**5 **(s13)
	
	**22**	**15.075**	**21**	**9 **(s25)	**5 **(s22)

**Subject_5 TCD-027**	**4**	**1.25301**	**3**	**0 **(s05)	**5 **(s02)
	
	**17**	**1.90167**	**9**	**8 **(s19)	**5 **(s05)
	
	**26**	**7.31028**	**21**	**10 **(s23)	**8 **(s23)

**Subject_6 NIMR-076**	**4**	**7.68038**	**2**	**0 **(s02)	**5 **(s02)
	
	**23**	**6.93772**	**7**	**6 **(s26)	**N/A**
	
	**35**	**8.37792**	**21**	**6 **(s38)	**17 **(s38)

**Subject_7 AHS-022**	**3**	**3.6903**	**21**	**8 **(s06)	**5 **(s03)
	
	**15**	**7.9402**	**19**	**10 **(s19)	**5 **(s15)
	
	**23**	**4.7744**	**20**	**6 **(s99)	**5 **(s23)

**Subject_8 AHS-012**	**4**	**3.69191**	**18**	**N/A**	**5 **(s04)
	
	**12**	**5.26977**	**19**	**5 **(s15)	**5 **(s12)
	
	**21**	**4.77444**	**21**	**6 **(s24)	**13 **(s21)

**Subject_9 AHS-011**	**4**	**8.56937**	**16**	**5 **(s09)	**5 **(s04)
	
	**15**	**21.3752**	**15**	**5 **(s18)	**5 **(s15)
	
	**25**	**28.0592**	**19**	**7 **(s25)	**5 **(s25)

**Subject_10 NIMR-029**	**5**	**50.5738**	**0**	**0 **(s08)	**25 **(s02)
	
	**24**	**75.5656**	**4**	**1 **(s21)	**N/A**
	
	**37**	**50.1098**	**14**	**9 **(s40)	**8 **(s30)

1) Fugl-Meyer, Hand section: range 0-21; 2) Motor Assessment Scale (MAS), "Hand movements" and "Advanced Hand Activities": maximum score: 0-12; 3) Stroke Impact Scale, hand function: range 5-25.

As the scores of the different scales changes, the values of the recorded mean force resultant vector change. This is mainly remarkable in the Fugl-Meyer scores.

Some preliminary results from a normal control subject and a pathological subject are here presented. The choice of the task and the sensors is based on the results of data mining algorithms applied to the pre-processed data [43].

Let's consider the task "Drinking" in a healthy control (male, 45 years old, right dominant hand, measurement of the left side) and in a pathological subject (male, 43 years old, right dominant hand, right side of hemiparesis, date of stroke 15/12/2005, measurement on the right side), 25 days and 131 days following the stroke onset. The number of samples from force measurements shown in Figure 6, 7 and 8 is 5400: as already stated, the sample frequency for data acquisition is 100 Hz, therefore the task lasts 5.4 seconds.

**Figure 9:**
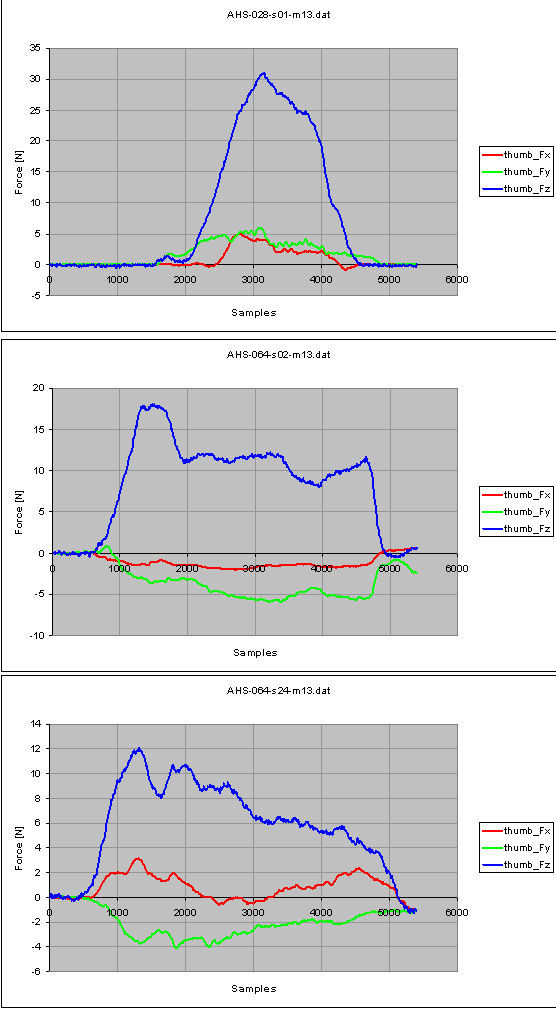
**Force measurements from the thumb, for the task 'Drinking': normal control subject (top); hemiparetic patient, 25 days after the stroke onset (middle) and 131 days after the stroke onset (bottom)**.

Figure 9 shows the force measurements from the thumb in the normal control (top plot), in the hemiparetic patient, 25 days following the stroke onset (middle plot) and 131 days following the stroke onset (bottom plot). In the healthy control, positive values in the Fx-direction can be observed. In normal circumstances, for a grasping movement, the thumb will be brought to the point where the index and the middle finger touch each other. In the diagnostic device, the thumb is fixated on the same height as the index finger. This causes a downwards movement of the thumb when the subject grasps the glass to drink. The positive values in Fy-direction means that the subject moves the thumb forwards when he positions the fingers around the glass to drink. The positive values for Fz-direction points out that the subject grasps the glass to drink. The force measurement from the thumb recorded 25 days following the stroke onset show negative values on the x-axis: the force is directed in the opposite direction than the motor performance in the normal control subject, pointing out that the subject moves the thumb upwards to bring the glass to the mouth, instead of moving downwards. The negative values observed along the Fy-direction mean that the subject pushes the thumb forwards to bring the glass to the mouth. The positive values along the z-axis allow to conclude that the subject tries and grasps the glass to drink. The force is exerted in advance than the normal control subject and it lasts till to the end of the attempt.

The force measurement from the thumb recorded 131 days following the stroke onset show the positive values on the x-axis, same direction as the motor performance in the healthy control, meaning that the subject moves the thumb downwards to bring the glass to the mouth, even if the force reaches the zero at about the half of the measurement, before still rising to positive values. The negative values observed along the y-axis point out that the subject pushes the thumb forwards to bring the glass to the mouth. The positive values along the z-axis mean that the subject tries and grasps the glass to drink. The force is exerted in advance than the normal control subject and it lasts till to the end of the attempt.

**Figure 10:**
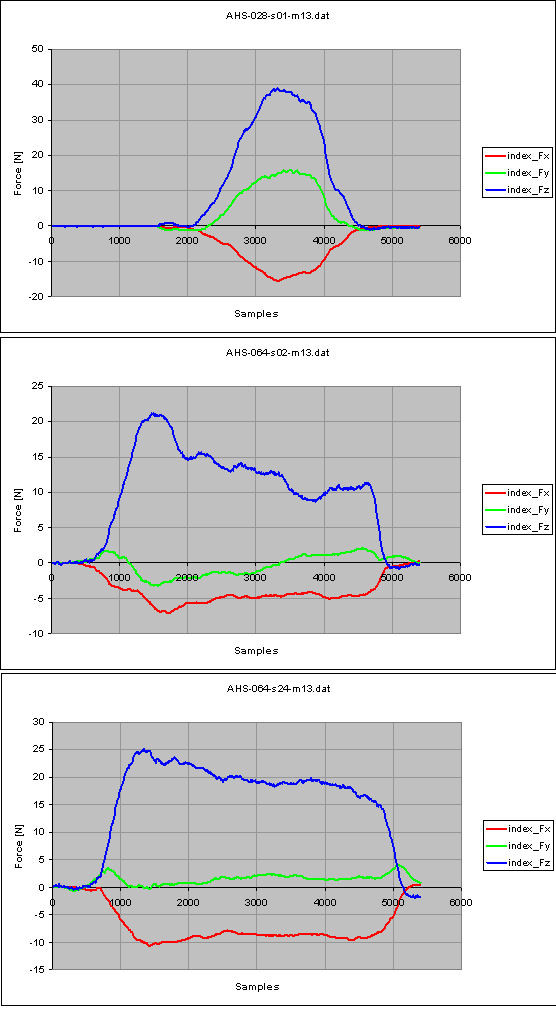
**Force measurements from the index finger, for the task 'Drinking': normal control subject (top); hemiparetic patient, 25 days after the stroke onset (middle) and 131 days after the stroke onset (bottom)**.

**Figure 11:**
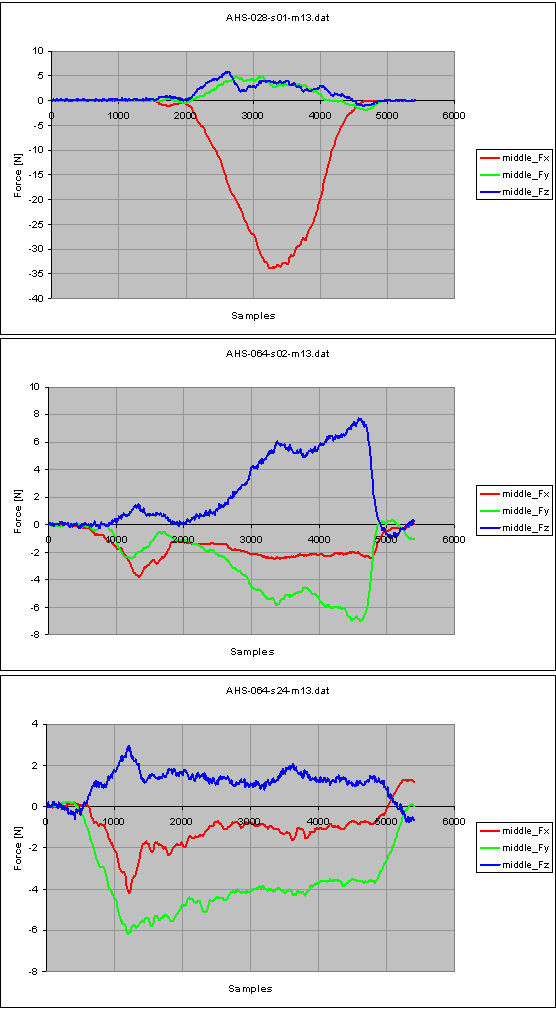
**Force measurements from the middle finger, for the task 'Drinking': normal control subject (top); hemiparetic patient, 25 days after the stroke onset (middle) and 131 days after the stroke onset (bottom)**.

Figure 10 and 11 show the force measurements in the normal control (top plot), in the hemiparetic patient, 25 days following the stroke onset (middle plot) and 131 days following the stroke onset (bottom plot) from the index finger and the middle finger respectively. Similar observations can be drawn for such measurements.

A large set of features characterizing the clinical recovery were extracted from the data according to the preliminary results from data mining techniques [42] in order to track the recovery process through milestones and to foresee the rehabilitation outcome through predictive markers: they are presented in a companion paper.

Finally, a study about the impact of the use of the platform, based on Health Technology Assessment (HTA) procedures, on care delivery was performed [44,45].

The study was aimed at outlining the epidemiological scenario, identifying the main factors of strengths and weaknesses in order to successfully manage the future commercialization of the diagnostic device and developing a theoretical model for the evaluation of the ADD costs and the potential costs savings. The results from this study show that HTA procedures provided inputs for bringing technical adjustments to the ADD and, at the same time, gave indications about its market potential: an overall acceptance of the proposed methodology was shown. On the whole, the results showed that the future common efforts should be aimed at defining a standard procedure for the assessment of functional recovery after stroke, in order to provide quantitative data for the interpretation of patients' conditions.

## Discussion and conclusion

Although following a neurological injury people might not be able to complete the actual everyday motions, they can either execute forces that closely represent the actual motion or at least some portion of these forces. The proposed device quantitatively assesses the severity of the injury and ultimately track the recovery process. It partially addresses the question whether the nervous system maintains a residual amount of control that is only hampered by weakness and other deficiencies in motor actuation, making it impossible for a person to complete these motions. The device allows the patients to practice forces at diminished or inappropriate levels. During the rehabilitation programmes they can progressively recover the levels where the real motion can be attempted.

Preliminary exemplary data on two subjects, an healthy and a chronic hemiparetic patient at two points in the recovery process, were presented: the recovery process therefore can be tracked as a recovery of the force patterns toward more normal.

The diagnostic device, which is has been validated in three different clinical centres in Europe, proved to be effective as a tool for experimental use in novel functional assessment procedures of post-stroke patients, according to the original specifications provided by medical doctors and therapists. The platform has also a range of other potential applications, from motor therapy (i.e. isometric exercise) to human-machine interface.

The ADD, which is the first device which acquires a great deal of different data (F/T data, clinical scales, natural language descriptions recorded by the physiotherapists) till now, can be also associated to a virtual reality environment for motor rehabilitation, as recently implemented in a device for isometric measurements in the hand derived from the Finger Device [46].

Results from a dedicated data mining approach showed that the platform can be also simplified in the future: a next version of the ADD should only include simpler force sensors at the thumb, the index, the middle finger and the seat. This effectively reduces the cost of the platform from eight to four sensors. The most important ADL tasks resulted "Drinking a glass", "Lifting a bag" and "Lifting a bottle".

The use of the diagnostic device associated with systems for brain imaging, such as Positron Emission Tomography (PET), functional Magnetic Resonance Imaging (fMRI), MagnetoEncephaloGraphy (MEG), near infrared spectroscopy (NIRS), and electroencephalography (EEG) will allow to monitor the degree of learning and the changes in motor performances induced by the rehabilitative treatments through traditional and robotic therapies.

The proposed platform could have also a notable potential impact for basic research in neuroscience, e.g. by comparing isometric performance of healthy controls and different patients, and for studying anticipative and high-level planning capabilities based on the study of whole-body dynamics in isometric conditions at the inception of voluntary movements.

Based on the presented results, the diagnostic device therefore shows wide potentials for the clinical practice, primarily based both on its effectiveness as a tool for functional assessment of post-stroke patients and its usability for the medical staff, and for basic research in neuroscience.

## Competing interests

The authors declare that they have no competing interests.

## Authors' contributions

SM participated in the design and the development of the device, data analysis and drafted the manuscript, AT and MM supervised the design and the development of the device, JVV coordinated the project, GC participated in the data analysis, SM and PD contributed to the scientific coordination, EG supervised the design and the development of the device, the data analysis and contributed to the manuscript preparation. All authors read and approved the final manuscript.
